# Electronic cigarette use among adolescents and young adults in Nigeria: Prevalence, associated factors and patterns of use

**DOI:** 10.1371/journal.pone.0258850

**Published:** 2021-10-22

**Authors:** Olufemi Erinoso, Afolabi Oyapero, Mary Amure, Moyosoore Osoba, Olatokunbo Osibogun, Kikelomo Wright, Akin Osibogun

**Affiliations:** 1 Department of Oral and Maxillofacial Surgery, Lagos State University Teaching Hospital, Lagos, Nigeria; 2 Department of Preventive Dentistry, Lagos State University College of Medicine, Lagos, Nigeria; 3 Department of Preventive Dentistry, Lagos State University Teaching Hospital, Lagos, Nigeria; 4 African Center of Excellence for Genomics of Infectious Diseases (ACEGID), Redeemer’s University, Ede, Osun State, Nigeria; 5 Department of Epidemiology, Robert Stempel College of Public Health and Social Work, Florida International University, Miami, Florida, United States of America; 6 Department of Community Health and Primary Health Care, Lagos State University College of Medicine, Lagos, Nigeria; 7 Department of Community Health and Primary Health Care, College of Medicine, University of Lagos, Lagos, Nigeria; Federation University Australia, AUSTRALIA

## Abstract

**Background:**

Electronic cigarettes (e-cigarettes) have emerged in the Nigerian market, and if used without supervision, may have damaging effects on the physical and mental health of users. Therefore, there is a need to determine the patterns of use, especially among adolescents and young adults. This study aims to assess the prevalence and factors associated with electronic cigarette use, as well as the relationship between their use and anxiety among adolescents and young adults in Lagos, Nigeria.

**Method:**

An online cross-sectional study among participants aged between 15–35 years. The survey had three sections: sociodemographic information, the pattern of e-cigarette use, and a 7-item Generalized Anxiety Disorder (GAD-7) scale. Bivariate and multivariable logistic regression analysis was used to identify factors associated with e-cigarette use. P-values <0.05 were considered significant. Statistical analysis was done using STATA-15.0 software.

**Results:**

Data from a total of 949 respondents was analysed. Participants had a mean age of 23.36 years (±3.97) and were predominantly female (55.64%). The prevalence of e-cigarette ever-use was 7.9% (95% CI: 5.8,10.0). Older age and being male were independently associated with higher odds of e-cigarette use. After adjusting for age and sex, alcohol use (p<0.001), friend’s use (p<0.001), and other tobacco product or substance use (p:0.05) remained significantly associated with higher odds of e-cigarette use. There was no association between anxiety levels and e-cigarette use.

**Conclusion:**

These findings suggest a higher likelihood of e-cigarette use among alcohol consumers, poly-tobacco or substance users and individuals with friends who use e-cigarettes. Health providers and policy makers in Nigeria might consider preventive measures aimed at young adults with the identified risk factors, as well as close monitoring of trends in e-cigarette use in the coming years.

## Introduction

Electronic cigarettes (e-cigarettes) are devices powered by batteries that provide vaporized nicotine and re-create the sensation derived from smoking conventional cigarettes without involving the combustion of tobacco [1, 2]. Modern e-cigarettes are more socially acceptable alternatives to combustible cigarettes among adolescents and young adults, due to their attractive design, user-friendly functions, less aversive smoking experiences, desirable flavors, and the ability to be used discreetly [3, 4]. E-cigarettes are increasingly becoming popular and may have deleterious effects on users by contributing to primary nicotine addiction amongst young adults and the renormalization of smoking behaviours [3, 4]. Further, some e-cigarettes contain nicotine, which has been associated with adverse mental health when used without supervision [5, 6].

Recent studies conducted on the prevalence of e-cigarettes have found a steady increase in use amongst young adults [4, 7, 8]. Among adolescents in the United States, there was nearly 14-fold increase in rates of current e-cigarette use among youth between 2011 and 2018, with e-cigarettes use exceeding combustible cigarette use; in 2018, past 30-day e-cigarette use was reported by 21% of high school (3.05 million) and 5% of middle school (570,000) students, compared with combustible cigarette use which was reported by 8% of high school (1.1 million) and 2% of middle school (200,000) students [7]. A similar increase has been reported in Europe [8]. In Africa, to our knowledge, few studies have explored the use of e-cigarettes. On the continent, knowledge levels of between 57% and 79% have been reported [9, 10], with a majority of ever users between ages 21–30 years, and a self-reported history of use not exceeding 12 months [11]. Other studies have also detailed the potentially deleterious effect of e-cigarette use in depressing long-term cessation [12]. Novelty and sensation seeking are probable reasons why young adults are predominantly drawn to using e-cigarettes [2].

As e-cigarettes have emerged in the Nigerian market [13], there is a need to monitor associated factors and patterns of use, especially among adolescents and young adults. Osibogun *et al*. [14] in an exploratory study of 20 young adults in Nigeria, detailed the perception of e-cigarettes as less harmful than combustible cigarettes, and seven participants reporting a history of ever using an e-cigarette. These findings indicate an awareness and use of e-cigarettes among youth in Nigeria. Nonetheless, there remains a dearth of literature on associated factors, the prevalence and pattern of use among adolescents and young adults.

Further, we identify a gap in the literature on the relationship with anxiety among adolescents and young adults in Nigeria, given the potential for adverse mental health effects from nicotine in e-cigarettes. The most serious concern regarding adolescent e-cigarette use is the potential for long-term effects on brain development and behavior [15]. Depressive symptoms have been associated with e-cigarette use generally and initiation of e-cigarette use in young people [16]. Although, Cummins *et al*. [17] documented associations between e-cigarette use and self-reported diagnosis of anxiety disorder, there is still a relative dearth of research on e-cigarette use among individuals with mental health conditions such as anxiety, a priority population with high overall smoking rates and at risk for profound tobacco-related health effects.

This study aims to assess the prevalence and factors associated with e-cigarettes use among adolescents and young adults in Lagos State, Nigeria. In addition, we investigate the relationship between e-cigarette use and anxiety among adolescents and young adults. Findings from this study can serve as baseline data for future intervention studies, and support regulatory policies aimed at reducing the overall burden of nicotine dependence among adolescents and young adults.

## Materials and methods

### Study design and settings

This was an online cross-sectional study conducted between December 2020 and February 2021 in Lagos State, Nigeria. Lagos State has a population of over 17 million people in 20 local government areas (LGAs) and 37 local council development areas consisting of approximately 2000 communities. It is the commercial nerve center and one of the most densely populated states in the country.

### Study participants and sampling method

Data was collected using an online sample of in-school adolescents and young adults within Lagos State aged between 15 to 35 years. A multi-stage sampling technique (a combination of simple random and cluster sampling techniques) was used. The simple random technique was used to select schools (secondary schools and Universities) within Lagos State and the cluster sampling for class groups within the selected schools.

A sampling frame of secondary and tertiary educational institutions in Lagos State was obtained from the Lagos State Ministry of Education website. Leaders of class groups within the selected educational institutions were contacted, and an online secured link was shared through WhatsApp and/ or email to members of class group networks. A convenient sample of students who gave consent proceeded to answer questions in the online survey. Members of the class groups who did not have access to the internet were contacted via telephone, and after consent, a telephone interview was conducted. The telephone interviews were conducted in English language. The data collection approach used was due to the social distancing measures being undertaken by the Federal government to curb the spread of COVID-19 disease.

### Data collection tool

An online survey was created using Survey Monkey, which is an online survey tool. A validated questionnaire adapted from the Global Youth Tobacco Survey (GYTS) version 1.2 2014 was used [18]. The questionnaire had three main sections, asking for information about [S1 Appendix]: Section A determined the baseline characteristics of the participants, including age, gender, education, and alcohol consumption. Section B. comprised ten questions about e-cigarette use, other tobacco product use, substance use, quit attempts, friends’ use of e-cigarette and use of other tobacco products. Section C comprised a 7-item Generalized Anxiety Disorder (GAD-7) scale (range, 0–21), which assessed self-reported symptoms and severity of anxiety respectively. The total scores of GAD-7 were categorized as normal (0–5), mild (6–10), moderate to severe (≥11) anxiety. The GAD scale has been validated in Nigeria using a large and diverse sample of the population [19].

### Statistical analysis

Demographic data was analyzed using descriptive statistics. Bivariate associations between variables were assessed using chi-squared test. Bivariate and multivariable logistic regression analysis using all the sociodemographic variables and anxiety levels (measured using the GAD scale) as independent variables to identify factors associated with e-cigarette use. P values <0.05 were considered significant. Statistical analysis was done using STATA 15.0 software (StataCorp LLC Lakeway Drive, College Station, Texas) [S2 and S3 Appendices].

### Ethics

Approval was obtained from the Health Research and Ethics Committee of the Lagos State University Teaching Hospital [LREC/06/10/1456]. Informed consent was obtained digitally from participants aged ≥18 years and informed consent from parents/guardians with assent was obtained from those between ages 15–17 years, prior to administration of the study questionnaire [S1 Appendix]. Participants (or their parents or guardians) indicated individual consent and/or assent by clicking on a button “I consent” or “I assent” on the first page of the online form after reading the study information, before gaining access to the main questionnaire [S1 Appendix]. The online mode of data collection was adopted by the research team because of the social and physical distancing public health measures in effect in Nigeria to curb the spread of the novel coronavirus disease 2019.

## Results

### Sociodemographic information

A total of 1006 participants were enrolled in the study, of which 949 (94.3%) completed the survey. Participants had a mean age of 23.36 years (± SD 3.97), and majority of the respondents were between the ages 18–24 years (61.9%). A male to female ratio of 1:1.25 was obtained within the study population [Table 1]. About 87.95% of participants had a college diploma or more, while 11.8% had obtained a secondary school diploma, and 0.2% had not completed their secondary school education. Twenty eight percent (27.9%) of respondents reported having a history of alcohol consumption “Table 1”.

**Table 1:** Descriptive variables and sociodemographic distribution of participants.

Variable	n (%)
Overall mean age [±SD]	23.36 [±3.97]
**Age**	n = 937
<18 years	23 (2.5)
18–24 years	580 (61.9)
>24 years	334 (35.7)
**Sex**	n = 949
Male	421 (44.4)
Female	528 (55.6)
**Educational level**	n = 946
Less than Secondary school	2 (0.2)
Secondary school	112 (11.8)
College and above	832 (88.0)
**Current alcohol consumption**	n = 944
Yes	263 (27.9)
No	681 (72.1)
**Ever Heard of E-cigarettes**	n = 875
Yes	522 (59.7)
No	353 (40.3)
**Where did you first see this image of E-cigarettes?**	n = 621
Traditional media	70 (11.3)
The internet	115 (18.5)
Social media	163 (26.3)
Friends	114 (18.4)
Social gatherings	94 (15.1)
Classroom	28 (4.5)
Other	37 (6.0)
**Ever used E-cigarettes**	n = 645
Yes	51 (7.9; 95% CI: 5.8,10.0)
No	594 (92.1)
**History of E-cigarette use**	n = 645
Current user (past 30-day use)	6 (0.9)
Former user (>30 days since last use)	45 (7.0)
Never user	594 (92.1)

Note: Traditional media: Television, radio, newspapers. The internet: online malls, news and non-news web pages. Social media: Facebook, twitter, Instagram, WhatsApp groups. Social gatherings: parties, lounges.

### Prevalence of electronic cigarette use and patterns of use

A majority of respondents had heard of e-cigarettes (59.7%), while 26.3% recognized the images of e-cigarettes from social media posts. Fifty-one (7.9%; 95% CI: 5.8,10.0) respondents reported ever using e-cigarettes, of which six (0.9%; 6/51) were current users (used e-cigarettes in the past 30days) and forty-five (7.0%) were former users (>30 days since last use) “Table 1”.

Among participants who had ever used e-cigarettes (n = 51), about 41.2% reported a lifetime use of between 2–10 days and 3.9% reported more than 50 days, with majority (n = 37, 72.6%) using it at home. Past 30-day use of e-cigarettes among respondents who reported ever using was 11.8% (n = 6). Furthermore, among e-cigarette ever-users (n = 51), about 9.8% (n = 5) reported an intention to quit, while 17.6% (n = 9) of 51 e-cigarette ever-users reported an actual quit attempt in the last 12 months “Table 2”.

**Table 2:** Pattern of use and quit attempt among E-cigarette users.

Variable	n (%)
**Life-time E-cigarette use**	n = 51
1 day	19 (37.3)
2–10 days	21 (41.2)
11–20 days	3 (5.9)
21–50 days	5 (9.8)
51–100 days	1 (2.0)
> 100 days	1 (2.0)
No response	1 (2.0)
**Past 30-day E-cigarette use**	n = 51
0 days	44 (86.3)
1–2 days	4 (7.8)
3–5 days	2 (3.9)
>6 days	0 (0)
No response	1 (2.0)
**Most common location of E-cigarette use**	n = 51
At home	37 (72.6)
At a restaurant	2 (3.9)
At a bar or club	9 (17.7)
Other	3 (5.9)
**Intention to Quit**	n = 51
Yes	5 (9.8)
No	5 (9.8)
No response	41 (80.4)
**Actual quit attempt in last 12 months**	n = 51
Yes	9 (17.6)
No	9 (17.6)
No response	33 (64.7)
**History of friend(s) who currently use e-cigarettes**	n = 847
Yes	247 (29.2)
No	584 (68.9)
I don’t know	16 (1.9)
**History of tobacco products or substance use**	n = 771
Yes	74 (9.6)
No	697 (90.4)

Note: Tobacco products: combustible cigarettes only (n = 46), cigar only (n = 1), water-pipe only (n = 1), smokeless tobacco only (n = 1), not reported (n = 18). Substance use: Cannabis/ marijuana (n = 6). Cigarettes and Water pipe (n = 1), Cigarettes and Cannabis (n = 1).

### Association between sociodemographic factors and self-reported anxiety (measured using the GAD scale)

E-cigarette use was reported predominantly by respondents above 18 years of age. Most participants had normal self-reported anxiety levels (62.1%), regardless of history of e-cigarette use (p>0.05). However, a greater fraction of e-cigarette ever users had normal anxiety levels compared to non-users (62.8% vs 62.0% respectively) “Table 3”.

**Table 3:** Association between E-cigarette use and generalised anxiety disorder.

GAD Level	Total	History of E-cigarette ever-use (%)	No History of E-cigarette ever-use (%)	p value
	n = 641 (100)	n = 51 (100)	n = 590 (100)	
Normal	398 (62.1)	32 (62.8)	366 (62.0)	0.85
Mild	100 (15.6)	9 (17.7)	91 (15.4)	
Moderate to Severe	143 (22.3)	10 (19.6)	133 (22.5)	
^**¶**^ **Age**	n = 635 (100)	n = 51 (100)	n = 584 (100)	
<18 years	15 (2.4)	0 (0)	15 (2.6)	0.18
18–24 years	374 (58.9)	26 (51.0)	348 (59.6)	
>24 years	246 (38.7)	25 (49.0)	221 (37.8)	

*p<0.05. GAD: Generalised Anxiety Disorder scale: GAD-7, normal (0–5), mild (6–10), moderate to severe (≥11) anxiety.

^**¶**^ Six participants declined to indicate their age.

A bivariate logistic regression model “Table 4” demonstrated that increased age in participants was associated with higher odds of self-reported e-cigarette use [95% CI: 1.01,1.14; p:0.02]. Similarly, female respondents had lower odds of self-reported e-cigarette use [95% CI: 0.21,0.71; p:0.002]. In addition, participants who reported consuming alcohol had eight times increased odds of self-reported e-cigarette use [95% CI: 4.17,15.97; p<0.001]; and likewise, participants who had friends who used e-cigarettes had higher odds of ever-use [OR: 5.05; 95% CI:2.98,8.57; p<0.001], as did participants who currently used other tobacco products and substances [OR:5.30; 95% CI: 2.65,10.59; p<0.001]. Anxiety levels were not significantly associated with e-cigarette use in the bivariate regression model (p>0.05).

**Table 4:** Factors associated with E-cigarette ever-use.

Variable	OR (95% CI)	p value	aOR (95% CI)	p value
**Age**	1.08 (1.01,1.14)	0.02*	1.05 (0.98,1.14)	0.18
**Sex**				
Male	1 (reference)		1 (reference)	
Female	0.38 (0.21,0.71)	0.002*	0.66 (0.32,1.36)	0.26
**Current alcohol consumption**				
No	1 (reference)		1 (reference)	
Yes	8.16 (4.17,15.97)	<0.001*	5.49 (2.58,11.70)	<0.001*
**History of friend (s) who currently use e-cigarettes**				
No	1 (reference)		1 (reference)	
Yes	5.05 (2.98,8.57)	<0.001*	4.43 (2.35,8.33)	<0.001*
**History of tobacco or substance use**				
No	1 (reference)		1 (reference)	
Yes	5.30 (2.65,10.59)	<0.001*	2.18 (1.00,4.76)	0.05*
**Educational level**				
High school and above	1 (reference)			
Less than Secondary school	0.26 (0.06,1.08)	0.06		
**Anxiety levels (GAD-7)**				
Normal	1 (reference)			
Mild	1.13 (0.52,2.45)	0.76		
Moderate to Severe	0.86 (0.41,1.80)	0.69		

*p<0.05. OR: odds ratio. aOR: adjusted odds ratio. GAD: Generalised Anxiety Disorder scale: GAD-7, normal (0–5), mild (6–10), moderate to severe (>11) anxiety. Pseudo R^2^ 0.24 for Multivariable logistic regression model.

In the multivariable logistic regression model “Table 4”, alcohol use [OR:5.49; 95% CI:2.58,11.70; p<0.001], a history of friends who used e-cigarettes [OR: 4.43; 95% CI:2.35,8.33; p<0.001], and current use of other tobacco products and substances remained significantly associated with e-cigarette use [OR:2.18 95% CI: 1.00,4.76; p:0.05].

## Discussion

The current study assessed the prevalence, factors associated with use and the relationship between e-cigarettes and anxiety levels in adolescents and young adults. We found that 7.9% of our study participants reported e-cigarette ever-use. We also found that increasing age, male sex, current alcohol consumption, ever-use of other tobacco products and substance (cannabis), as well as friend’s use of e-cigarettes, are associated with higher odds of e-cigarette ever-use. In addition, our findings demonstrated no statistically significant association between e-cigarette ever-use and anxiety levels, although a greater proportion of e-cigarette ever-users had normal anxiety levels compared to non-users.

The prevalence of e-cigarette use is well documented in the United States and Europe. In the United States, the prevalence of current e-cigarette use was estimated at 3.2% in 2018 [20]. While in Europe, prevalence rates of ever-use of e-cigarettes amongst persons aged 15years and above were estimated at 11.6% in 2014 [8]. Few studies have assessed the prevalence of e-cigarette use in Africa. About 2.71% of South-African adults (~1.09 million people), used e-cigarettes daily or occasionally in 2018 [21]. Osibogun *et al*. [14] in an early exploratory study reported a prevalence of 35% in a sample of 20 participants in Nigeria. Our findings indicate a prevalence of 7.9% ever-users and 0.9% current users. These findings may suggest a relatively lower prevalence in Africa compared to Europe and the United States. The relatively high cost of e-cigarettes may account for the lower prevalence, as well as the new entrance of these products into the market. However, if unchecked this trend may rise because while e-cigarettes are less harmful than combustible tobacco products [22], the long-term effects of use are unknown, and evidence is accumulating on the adverse health effects of e-cigarettes [4, 23]. Also, with increasing marketing and access to e-cigarette shops, the use of e-cigarettes can rise correspondingly [24]. The implication of this could be a higher prevalence in the future, and e-cigarettes serving as gateway products for young adults to progress to combustible tobacco use [4, 16], leading to a concurrent increase in tobacco-induced diseases.

In this study young adults above 18 years were more likely to use e-cigarettes than adolescents between 15 and 18 years. Similarly, more males than females used e-cigarettes, with significantly higher odds of ever-use among males than females. Our findings are consistent with earlier reports from the United States and Nigeria [14, 25, 26]. Other studies have also described the moderating effect of gender on the link between the ever-use of e-cigarettes and smoking status, with significantly lower odds of e-cigarette use among females [27]. This phenomenon may be explained by the social perception towards smoking that is generally more tolerant of male than female smokers [28], as well as the common social disapproval of women smoking in many African societies [29]. The more common use among adults may be due to current policies prohibiting smoking among adolescents [30], affordability, and the higher exposure to advertising among young adults over time [28]. Therefore, targeted health warning and messaging based on gender differences and age is crucial in mitigating the uptake of e-cigarettes, especially among adolescent and young adult never-users in the country.

Respondents that self-reported current alcohol consumption had five times significantly higher odds of e-cigarette ever-use, while those with a history of ever-using tobacco products or substances had four times significantly higher odds. Corroborating our findings, several studies have reported a positive association between e-cigarette use and concurrent alcohol [31], combustible cigarette [32] and cannabis use [6, 25, 26, 33–35]. The direction of the relationship between e-cigarettes, alcohol use, other tobacco products and cannabis use is still unclear. Nonetheless, Bold and colleagues [36] detailed a unidirectional association between e-cigarette use and future cigarette use. To support their findings, some evidence suggests that the developing brain in adolescents and young adults are very sensitive to the rewarding effect of nicotine [36, 37], therefore use of e-cigarettes may lead to early dependence on nicotine and increase the risk of transitioning to other tobacco products [36]. Further, the phenomenon of concurrent use, seen in the present study, could be explained by the similar social profile shared by e-cigarette users, tobacco, cannabis and alcohol users [34]. Longitudinal and experimental studies will be necessary to clarify the complex and potentially bidirectional relationship between e-cigarette use, alcohol, polytobacco and substance use among adolescents and young adults in the country [38]. Future studies can explore this relationship and identify which of these factors, if any, are drivers of e-cigarette use among adolescents and young adults.

Consistent with other studies [39–41], our results suggest that e-cigarette users with friends who were current users of e-cigarettes had four times significantly higher odds of e-cigarette ever-use. The link between the ever-use of e-cigarette and friends’ e-cigarette use may mean adolescents and young adults who perceive greater social approval are more likely to use e-cigarettes [39]. In addition, our finding may be explained by the processes- influence and selection [41]. ‘Influence’ is an inclination of individuals to adopt the behaviour of their peers, for example, e-cigarette use. On the other hand, with ‘selection’ individuals tend to choose peers who have similar behaviours as themselves, such as e-cigarette use [41, 42]. Wallace *et al*. [41] in a survey of college students found that having friends who used e-cigarettes was positively associated with the following: likelihood of having been offered an e-cigarette by a friend previously, the perceived likelihood of taking this offer in the future, as well as e-cigarette use. Therefore, careful consideration must be given to young adults with a history of alcohol, tobacco product, substance use, and a social network of e-cigarette users during screening exercises for at-risk nonsmokers and cessation counselling for e-cigarette users with the intention to quit.

In January 2006, Nigeria became a Party to the WHO Framework Convention on Tobacco Control (FCTC) and began developing supporting policies aligned with the FCTC articles [30]. Currently, the prevalence of tobacco use is estimated at 5.6% in individuals above 15 years [43]. The British American Tobacco (BAT) company holds a significant market share nationwide [44], and in the past, claims to have partnered with government agencies on tobacco regulatory policies [45]. Tobacco control policies in the country at present ban advertising and promotional activities from tobacco manufacturers and retailers, however, a loophole in the law permits advertising activities to consenting adults [30]. In addition, except in designated smoking areas, all forms of smoking are prohibited in indoor public places and outdoor social venues and restaurants [30].

Presently, there are no restrictions to the purchase, sale, and advertising of e-cigarettes, according to the National Tobacco Control Regulations, 2019 [30, 46]. Similarly, there are no regulations surrounding the contents and labelling of these products. The lack of policies regulating e-cigarettes can potentially allow the tobacco industry to freely market these products, encouraging unintended effects that can be compounded by their inherent novelty, flavours, and addictive nicotine content. A review of e-cigarette products at a major online retail store in Lagos indicates the presence of 2nd to 4th generation devices at prices ranging from 4500 naira (~$12) to 50,000 naira (~$132) [13]. These prices suggest e-cigarettes are more expensive than combustible cigarettes (a pack ~400 naira, $1.50c). Nonetheless, they remain available and affordable for a significant proportion of the population in Lagos, especially those of middle to upper socioeconomic status. The results from this study can provide evidence for revising the National Tobacco Control Regulations to capture e-cigarettes or support the design and implementation of a State-wide policy aimed at regulating e-cigarettes in Lagos, Nigeria.

The present study provides early evidence on the prevalence and factors associated with e-cigarette use in Nigeria. Nonetheless, we identify several limitations. The cross-sectional design of the study does not permit the inference of a temporal or causal relationship between e-cigarette use, sociodemographic factors and anxiety. The data from respondents were self-reported and not directly measured. Therefore, the possibility of a social-desirability bias for some of the questions that relate to cannabis and combustible cigarette use, especially among adolescents, cannot be discounted in a relatively socially conservative society like Nigeria. In addition, information on the age of initiation, as well as the type of e-cigarette device, daily number of puffs, the presence or absence of flavours and quantity of nicotine were not collected. This information could help identify the frequency of use and the particular type of e-cigarettes commonly used. Furthermore, our study results may not be generalizable to adolescents and young adults who are not attending schools. Lastly, data collection was online and in the English language. Therefore, these factors might have served as a barrier for individuals who could not communicate in English and did not have internet access, thereby limiting the generalizability of our findings.

## Conclusion

Our findings suggest a prevalence of 7.9% for e-cigarette ever-use, and 0.93% for current use (past 30-day). Further, a higher likelihood of e-cigarette use was also found with increasing age, males, current alcohol users, concurrent polytobacco and substance users, and respondents with friends who use e-cigarettes. On the other hand, we found no significant association between anxiety levels and e-cigarette use. Policies to limit the access and use of e-cigarettes among adolescents and non-smokers are encouraged. Findings from this study can help inform health policymakers about the magnitude and characteristics of e-cigarette–users. This information can also facilitate monitoring of trends, guide tobacco regulatory policy, and tobacco control campaigns in the country.

## Supporting information

S1 Appendix Survey questionnaire. (TXT)

S2 Appendix E-cig.NGR_dataset. (DOCX)

S3 Appendix E-cig.NGR_Do-file. (XLS)
