# Supplementary material for: Electronic cigarette use among adolescents and young adults in Nigeria: Prevalence, associated factors and patterns of use
Source: PLoS One. 2021 Oct 22;16(10):e0258850. doi: 10.1371/journal.pone.0258850 (PMC8535460; doi:10.1371/journal.pone.0258850)
Supplement: S2 Appendix — (DOCX) [file pone.0258850.s002.docx]

**Appendix**

# **Data Collection Tool and Data Code**

**Section A:** Demographic information

A.1 Age in years (at last birthday): ---------

[AgeCategory: a. <18 (0). B. 18-24 (1). C. >25 (2)]

A.2. Sex: a) M (0 ) b) F (1 )

A.3 Do you consume Alcohol (*in the last 90 days*) a) Yes (1) b) No (0)

A.4 What is your highest level of Education?

| Primary school 2 | Secondary school 1 | University 0 | Post-graduate 0 |
| --- | --- | --- | --- |

**Section B.** The next questions ask about electronic cigarettes.

B1. Before today, had you ever heard of electronic cigarettes or e-cigarettes?

a. Yes [1]

b. No [0] (**If No to B1. Skip to Section C**)

B2. Where did you first hear about electronic cigarettes?

1. Traditional media: Television, radio, newspapers? 0
2. The internet (online malls, news and non-news web pages)? 1
3. Social media (Facebook, twitter, Instagram, WhatsApp groups)? 2
4. Friends? 3
5. Social gatherings (parties, lounges)? 4

B3. Have you ever used an electronic cigarettes or e-cigarettes?

a. Yes [1]

b. No [0] **(If No to B2. Skip to Question B.9)**

B4. During past 30 days, on how many days did you use electronic cigarettes?

a. 0 days [0]

b. 1 or 2 days [1]

c. 3 to 5 days [2]

d. 6 to 9 days [3]

e. 10 or 19 days [4]

f. 20 to 29 days [5]

g. All 30 days [6]

B5. In total, for how many days have you used an electronic cigarette or e-cigarette in your entire life?

a. 0 days [0]

b. 1 day [1]

c. 2 to 10 days [2]

d. 11 to 20 days [3]

e. 21 to 50 days [4]

f. 51 to 100 days [5]

g. More than 100 days [6]

B6. Do you want to stop smoking electronic cigarettes now?

a. I have never smoked electronic cigarettes [3]

b. I don’t smoke electronic cigarettes anymore [2]

c. Yes [1]

d. No [0]

B7. During the past 12 months, did you ever try to stop smoking electronic cigarettes?

a. I have never smoked electronic cigarettes [3]

b. I did not smoke electronic cigarettes during the past 12 months [2]

c. Yes [1]

d. No [0]

B8. The last time you smoked electronic cigarettes during the past 30 days, where did you smoke it? (SELECT ONLY ONE RESPONSE)

a. I did not smoke electronic cigarettes during the past 30 days [0]

b. At home [1]

c. At a restaurant [2]

e. At a bar or club [3]

f. Other. [Please specify: …………………..] [4]

B9. Do any of your friends smoke electronic cigarettes?

a. None of them [0]

b. Some of them [1]

c. Most of them [2]

d. All of them

B10. Have you ever used any tobacco product (e.g cigarettes, smokeless tobacco) asides electronic cigarettes and shisha?

a. Yes [1]

b. No [0]

B10b. If Yes to E1. Please specify the tobacco product (s): ……………………………………..

**Section C**: General Anxiety Depression (GAD-7) Scale

C1: Total GAD Score

| **Over the last 2 weeks, how often have you been bothered by the following problems?**  Use **“X”** to indicate your answer**.** | Not always | Several days | More than half the days | Nearly every day |
| --- | --- | --- | --- | --- |
| 1. Feeling nervous, anxious or on edge | 0 | 1 | 2 | 3 |
| 2. Not being able to stop or control worrying | 0 | 1 | 2 | 3 |
| 3. Worrying too much about different things | 0 | 1 | 2 | 3 |
| 4. Trouble relaxing | 0 | 1 | 2 | 3 |
| 5. Being so restless that it is hard to sit still | 0 | 1 | 2 | 3 |
| 6. Becoming easily annoyed or irritable | 0 | 1 | 2 | 3 |
| 7. Feeling afraid as if something awful might happen | 0 | 1 | 2 | 3 |

C1Category: normal (0-5), mild (6-10), moderate to severe (>11) anxiety.
